# *Shallot virus X* p42 Protein Expressed in Concert with Virus Movement Proteins Is a Suppressor of Two Plant Antiviral Defense Mechanisms

**DOI:** 10.3390/plants14162552

**Published:** 2025-08-16

**Authors:** Denis A. Chergintsev, Alexander A. Lezzhov, Ekaterina A. Lazareva, Anastasia K. Atabekova, Anna D. Solovieva, Sergey Y. Morozov, Andrey G. Solovyev

**Affiliations:** A. N. Belozersky Institute of Physico-Chemical Biology, Moscow State University, 119992 Moscow, Russia; ledumpalustre86@gmail.com (D.A.C.); lezzhov-genetic@mail.ru (A.A.L.); lazareva-katrina@mail.ru (E.A.L.); asya_atabekova@mail.ru (A.K.A.); l_anna2000@mail.ru (A.D.S.); morozov@genebee.msu.ru (S.Y.M.)

**Keywords:** plant virus, plant defense response, RNA silencing, nonsense-mediated decay, virus protein, silencing suppressor, RNA binding, microtubules, expression mechanism, leaky scanning

## Abstract

The genomes of viruses in the *Allexivirus* genus encode the p42 protein, which is considered the hallmark of the genus. The functions of p42 have not yet been studied experimentally and cannot be predicted based on sequence similarity, as p42-related proteins are not found among known cell or viral proteins. Here, p42 of *Shallot virus X* (ShVX), the type allexivirus, is demonstrated to be translated via a leaky scanning mechanism on a template comprising three “triple gene block” (TGB) transport genes and the p42 gene. Sequence analysis shows that this p42 expression mechanism is conserved in the vast majority of allexiviruses. p42 binds single-stranded RNA (ssRNA) but not double-stranded RNA (dsRNA) in vitro and localizes to the cytoplasm in association with microtubules and microtubule-bound bodies. In transient expression assays, p42 exhibits weak but detectable suppression of silencing induced by ssRNA but not by dsRNA. In addition, p42 suppresses silencing in the context of virus infection. Furthermore, p42 inhibits nonsense-mediated RNA decay (NMD) induced by a long 3′-terminal untranslated region of mRNA. Taken together, these findings provide initial evidence that the ShVX TGB/p42 gene module functions as a single genomic unit in terms of protein expression, that p42 acts as a suppressor of NMD and silencing, and that it may have multiple roles, while the precise biological significance of p42 in these roles remains to be experimentally confirmed.

## 1. Introduction

In general, virus genomes contain two essential gene modules, namely, the replicative module, encoding one or more proteins required for genome replication, and the structural module, coding for one or more components of the viral capsid [1]. The genomes of positive-stranded RNA plant viruses range from four to nineteen thousand nucleotides (nt) in length [2,3]. In small genomes, the replicative and structural modules comprise a substantial portion of genomic RNA. For example, in the *Turnip crinkle virus* (TCV) genome, which is 4054 nt in length, these two modules occupy 83.4%, while in the *Tobacco mosaic virus* (TMV) genome (6395 nt in length) —88.4%. The remainder of the genome in TCV, TMV, and viruses of similar size encodes another essential module, which is specific to plant viruses and absent in viruses that infect hosts in other kingdoms of life. This transport module encodes one or more so-called movement proteins (MPs), which enable the transport of viral genomes through plasmodesmata, the channels that interconnect cells in plant organisms, thus providing a basis for the symplastic nature of plant tissues and serving as routes for the transport of macromolecules between plant cells [4,5,6,7].

Genomes of larger size, besides the three gene modules essential for plant viruses, contain additional genes encoding proteins with auxiliary functions. Among those, the most widespread are viral suppressors of RNA silencing (VSRs), which are structurally and functionally diverse proteins that inhibit the antiviral plant response by targeting different components of RNA silencing machinery, binding viral double-stranded replication intermediates or virus-specific small interfering RNAs (siRNA), or blocking the cell-to-cell transport of silencing signal [8,9,10,11]. To date, VSRs have been identified for most studied plant viruses, suggesting the importance of this virus-encoded function [9,11]. Indeed, deletion of the silencing suppressor gene has been shown to lead to an inability of the virus to establish a systemic infection in plants [12,13]. Since VSRs are essential for RNA virus infection in plants, viruses with small genomes that lack the coding capacity for dedicated VSRs have evolved such that proteins of structural or replicative modules have acquired the additional VSR function. This function has been demonstrated, for example, for the TMV 126-kDa replicase component and the TCV capsid protein (CP) [14,15,16,17,18,19].

In addition to well-characterized genes that code for proteins with known functions, the genomes of many plant viruses contain open reading frames (ORFs) that encode proteins whose functions have not yet been determined experimentally and cannot be inferred from their sequence, as they lack similarity to known viral or cell proteins. One such protein, termed p42, is encoded by the genome of *Shallot virus X* (ShVX) [20].

ShVX is the type species of the genus *Allexivirus* in the *Alphaflexiviridae* family. Like other viruses in this genus, ShVX is a latent virus and does not induce visible symptoms in infected plants [21]. In naturally infected plants, ShVX and other allexiviruses are often found alongside other co-infecting viruses, mainly from the *Potyvirus* and *Carlavirus* genera [21,22], suggesting that ShVX may be part of virus associations. It is worth noting that allexiviruses can be transmitted by mites [23]. The ShVX genome is single-stranded RNA (ssRNA) of positive polarity that is 8832 nt long, excluding the 3′-terminal poly(A) tail [20]. The ShVX genome encodes seven proteins. The replicative module of the ShVX genome is represented by the 5′-proximal gene that codes for a 196 kDa protein. According to sequence analysis, this protein includes methyltransferase, AlkB (Alkylation B), helicase, and RNA-dependent RNA polymerase domains [24]. Downstream of the replicase gene, the ShVX genome contains a transport module, which consists of three genes and is therefore termed as “triple gene block” (TGB) [20,25]. As studied for other viruses, the TGB-encoded proteins TGB1, TGB2, and TGB3 act together to direct the viral RNA genome to and through PD channels, linking replication and cell-to-cell transport of viral genome [25,26,27,28]. The 3′-proximal region of the ShVX genome includes a virus structural module represented by a single gene of virus CP required for the formation of flexuous filamentous ShVX virions and the gene encoding a cysteine-rich protein (CRP) [20]. As shown for other allexiviruses, such as *Garlic virus X* and *Garlic virus B* (GarV-B), CRP has the VSR function, which requires protein localization to the cytoplasm [29,30]. Interestingly, CPs of GarV-B and *Garlic virus D* are also VSRs, and the GarV-B CP and CRP can inhibit the VSR activity of each other [30].

An additional gene, located between the transport and structural modules of the ShVX genome, encodes the p42 protein, which is considered the hallmark of the *Allexivirus* genus [20]. Proteins exhibiting sequence similarity to p42 are found neither in other viruses nor among cell proteins. The expression of p42 in ShVX-infected shallot and onion plants has been demonstrated by Western blotting [31]. Although some observations indicate an association of p42 with ShVX virions and/or virion formation [32], these data have not been verified. Therefore, the function of p42 remains unknown. It should be noted that the p42 functional analysis is greatly impeded by the absence of an infectious ShVX cDNA clone, which would enable a reverse genetic approach to studying protein functions in the context of viral infection.

Positive-stranded RNA virus genomes usually contain multiple genes; however, in eukaryotes, only the 5′-proximal gene can be translated on such templates. In alpha-like viruses, expression of other 5′-distal genes typically requires subgenomic RNAs (sgRNAs). These sgRNAs are 3′-coterminal with the genomic RNA and have 5′-termini located upstream of the start codons of the individual genes [33]. Although sgRNAs can comprise several genes, they are functionally monocistronic because only their 5′-proximal gene is translated. Among exceptions from this rule, a well-studied example is TGB, the transport genomic module, which is present in the ShVX genome and in the genomes of many viruses of the orders *Martellivirales*, *Tymovirales*, and *Hepelivirales*. Initial studies of *Potato virus X* (PVX; the *Potexvirus* genus) indicated that the three TGB proteins are expressed from two sgRNAs, one of which serves as a template for TGB1 translation, while another directs synthesis of TGB2 and TGB3, with the TGB3 translation being initiated by a leaky scanning mechanism [34,35]. More recent analysis of viruses in the *Potexvirus*, *Carlavirus*, and *Lolavirus* genera has clearly demonstrated that the three TGB proteins are translated from a single sgRNA and that TGB2 and TGB3 are expressed via leaky scanning [36]. In most allexiviruses, the TGB3 ORF lacks an AUG initiator codon [20,37]. Using mutational analysis, the translation of ShVX TGB3 has been shown to be initiated at a non-canonical initiator codon ACG via a leaky scanning mechanism [37]. The expression of other 5′-distal genes in the ShVX genome has not yet been studied.

Here, we show that ShVX p42 is translated on a tetracistronic RNA that contains the TGB and p42 genes via a leaky scanning mechanism. In in vitro experiments, p42 is shown to bind RNA. GFP-fused p42 localizes to the cytoplasm in association with microtubules and microtubule-associated bodies. Furthermore, the p42 protein is shown to suppress RNA silencing and nonsense-mediated RNA decay in plant cells.

## 2. Results

### 2.1. Expression of ShVX p42 Gene

**Figure 1:**
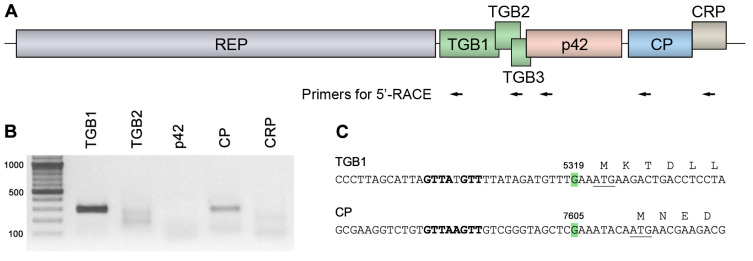
5′-RACE analysis of ShVX-specific sgRNAs. (**A**) Schematic representation of the *Shallot virus X* genome, showing the positions of the primers used for 5′-RACE, indicated by arrows. Each arrow corresponds to two primers used for the first and second rounds of amplification. (**B**) Products of 5′-RACE analyzed in an agarose gel. Size markers are shown in the left lane. The expected size of the 5′-RACE products is approximately 300 base pairs in all cases. (**C**) Mapped 5′-termini of TGB1 and CP sgRNAs. The 5′-terminal residues are shown in green, and their positions in the ShVX genomic RNA are indicated. The beginning of the TGB1 and CP coding sequences is shown; the initiator codon is underlined. The sequence element corresponding to the consensus sequence of sgRNA promoters in the *Alfaflexiviridae* family, GTTAAGTT, is shown in bold.

The expression of p42 encoded by genomes of ShVX and other allexiviruses has not yet been studied. In general, the expression of 5′-distal genes in ShVX can be expected to involve a number of sgRNAs that can serve as templates for translation, as demonstrated for other viruses of the *Alphaflexiviridae* family [36]. To analyze ShVX-specific sgRNA, 5′-RACE (rapid amplification of cDNA ends) was carried out using total RNA isolated from ShVX-infected shallot plants and a set of primers consisting of five pairs located downstream of the TGB1, TGB2, p42, CP, and CRP genes (Figure 1A). The ShVX TGB3 was not included in the analysis, as it was shown to be translated by a leaky scanning mechanism on a template containing both TGB2 and TGB3 genes [37]. The 5′-RACE analysis revealed distinct amplification products only for the TGB1 and CP genes (Figure 1B). To map the 5′-terminal residues of the TGB1 and CP sgRNAs, the amplification products were cloned, and five independent clones were sequenced for each gene. For both TGB1 and CP, the analyzed clones were identical in their sequence. As a result, the 5′-terminal residues of the TGB1 and CP sgRNAs were mapped to G residues in positions 5319 and 7605, respectively (Figure 1C).

**Figure 2:**
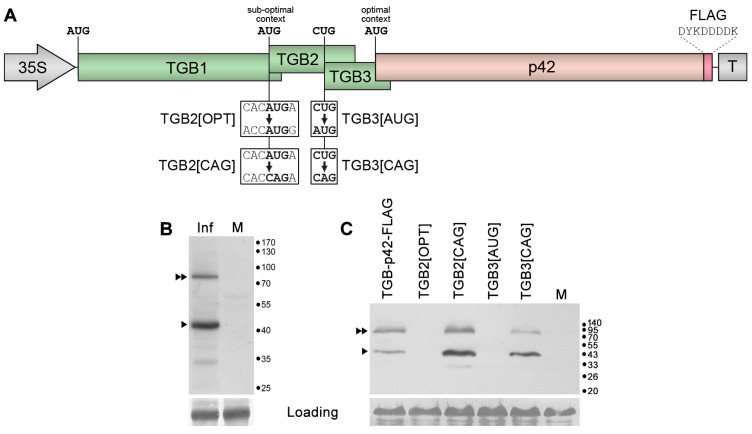
Analysis of the mechanism of p42 expression. (**A**) Schematic representation of the TGB-p42-FLAG construct. Genes are shown as boxes. The positions of the 35S promoter and the 35S terminator (T) are shown. The position and amino acid sequence of the FLAG epitope fused to p42 are indicated. Below the schematic, mutations introduced into TGB-p42-FLAG are shown, and the names of the respective mutants are indicated. (**B**) Western blot analysis of p42 accumulation in leaves of *N. benthamiana* agroinfiltrated for expression of the TGB-p42-FLAG construct using FLAG-specific antibodies. Inf, agroinfiltrated leaf. M, mock (leaves infiltrated with a buffer). The positions of p42 and its putative dimer are indicated by a single triangle and a double triangle, respectively. The positions of the molecular mass markers are shown on the right. (**C**) Influence of the introduced mutations on p42 translation. Accumulation of wt and mutant proteins was detected by Western blotting with FLAG-specific antibodies. M, mock-inoculated leaf. The positions of p42 and its putative dimer are indicated by a single triangle and a double triangle, respectively. The positions of the molecular mass markers are shown on the right.

**Figure 3:**
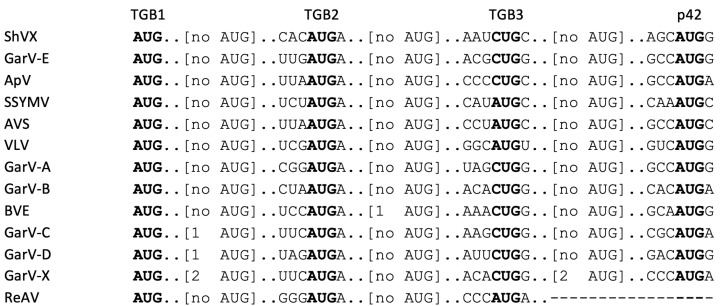
The contexts of initiator codons and presence of internal AUG triplets in the TGB/p42 region of allexivirus genomes. The contexts of the TGB2, TGB3, and p42 initiator codons are shown. The number of AUG triplets between the initiator codons of TGB1, TGB2, TGB3, and p42 is indicated in square brackets. Dashes indicate the absence of the p42 gene in the ReAV genome. The following viruses were included in the analysis: ShVX, *Shallot virus X* (accession number NC_003795.1); GarV-E, *Garlic virus E* (NC_004012.1); ApV, *Arachis pintoi virus* (NC_032104.1); SSYMV, *Senna severe yellow mosaic virus* (NC_076419.1); AVS, *Alfalfa virus S* (NC_034622.1); VLV, *Vanilla latent virus* (NC_035204.1); GarV-A, *Garlic virus A* (NC_003375.1); GarV-B, *Garlic virus B* (NC_025789.1); BVE, *Blackberry virus E* (NC_015706.1); GarV-C, *Garlic virus C* (NC_003376.1); GarV-D, *Garlic virus D* (NC_022961.1); GarV-X, *Garlic virus X* (NC_001800.1); ReAV, *Rehmannia allexivirus* (PP097219.1).

The absence of a 5′-RACE amplification product for the p42 gene (Figure 1B) suggests that p42 expression does not require a specific sgRNA. In this case, p42 translation could occur on a longer sgRNA, such as TGB1 sgRNA, via a leaky scanning mechanism or internal ribosome entry. A sequence analysis of the region including the TGB and p42 genes in the ShVX genome revealed that (1) the p42 gene overlaps the upstream TGB3 gene; (2) the TGB2 initiator codon is in a suboptimal context according to Kozak [38,39] (Figure 2A); (3) the TGB2 initiator AUG codon is the only AUG triplet in the region from the beginning of the TGB1 gene to the p42 initiator AUG codon; and (4) the p42 initiator AUG codon is in an optimal context for translation initiation (Figure 2A). These observations were compatible with the hypothesis that translation of p42 occurs on the template of TGB1 sgRNA by leaky scanning. In this process, a scanning ribosome subunit that has bypassed the TGB1 initiator codon can also bypass the suboptimal TGB2 AUG and the inefficient TGB3 CUG initiator codon; this allows the ribosome to reach the next AUG triplet, which is the p42 initiator codon (Figure 2A).

To verify this hypothesis, a region of the ShVX genome comprising TGB and the p42 gene was subcloned into a binary vector under the control of the 35S promoter. During the cloning, a short sequence encoding the eight-amino-acid FLAG epitope [40] was added in frame with the p42 ORF. The resulting construct, named TGB-p42-FLAG (Figure 2A), was used to transform *Agrobacterium tumefaciens* cells. The resulting bacterial clone was used for agroinfiltration of *Nicotiana benthamiana* leaves. Analysis of the leaves by Western blotting with FLAG-specific antibodies carried out at 3 days post infiltration (dpi) revealed a major band of size expected for p42-FLAG (approx. 43 kDa) and an additional band of approx. 85 kDa that likely represent a p42 dimer (Figure 2B). An additional faint band of 33 kDa found in the infiltrated leaves but not in the control could be a product of partial p42 proteolysis (Figure 2B). Therefore, these data show that p42 can be translated on the template of tetracistronic mRNA.

To determine whether p42 translation on this template occurs via leaky scanning, a series of TGB-p42-FLAG mutants was generated. These included TGB2[OPT], in which the context of the TGB2 AUG was made optimal according to Kozak [38,39]; TGB2[CAG] with the TGB2 AUG replaced by CAG, which has never been reported as an alternative initiator codon; TGB3[AUG], in which the alternative TGB3 initiator codon CUG was replaced by AUG; and TGB3[CAG], with the TGB3 initiator replaced by the non-initiator codon CAG (Figure 2A). Agrobacterial cultures carrying the mutant constructs and the control wild-type (wt) construct TGB-p42-FLAG were infiltrated into *N. benthamiana* leaves. Western blot analysis of infiltrated leaves with FLAG-specific antibodies revealed that the mutations introduced to generate TGB2[OPT] and TGB3[AUG] that increased the efficiency of translation initiation of TGB2 and TGB3, respectively, blocked the synthesis of p42 (Figure 2C). On the other hand, blocking of TGB2 and TGB3 translation in mutants TGB2[CAG] and TGB3[CAG] resulted in increased p42 levels, with the TGB2 initiator codon mutation having a more pronounced effect (Figure 2C). These data show that the p42 translation on mRNA mimicking the TGB1 sgRNA depends on translation initiation of the two upstream genes, excluding, therefore, internal ribosome entry as a mechanism of p42 translation. Moreover, p42 translation is only possible when translation initiation of the two upstream genes is suppressed, and it is blocked when translation of at least one of these genes is initiated efficiently. Collectively, these data demonstrate that p42 is translated on the template of TGB-p42-FLAG-derived tetracistronic RNA via a leaky scanning mechanism. Based on this conclusion, we suggest that the TGB1 sgRNA is the template for p42 translation in natural ShVX infection.

### 2.2. Possible Mechanism of p42 Expression in Other Allexiviruses

The leaky-scanning expression of ShVX p42 from the TGB1 sgRNA requires a weak TGB2 initiator, an inefficient TGB3 initiator, and the absence of other initiator codons in the TGB region. To determine whether this expression mechanism is universal for the *Allexivirus* genus, we analyzed the conservation of these features in the genomes of currently known allexiviruses. Initial analysis revealed that the initiator codons previously predicted for the TGB1 genes of four allexiviruses were located upstream of the 5′-terminal TGB1 sgRNA residues predicted by sequence alignment to the ShVX sequence (Supplementary Figure S1). When the initiator codons located downstream of the predicted 5′-terminal TGB1 sgRNA residues were taken as the true TGB1 initiators in these viruses (Supplementary Figure S1), it appeared that seven other allexiviruses, in addition to ShVX, had the above-listed features of the TGB/p42 genomic region (Figure 3) that enable p42 expression by the leaky scanning mechanism.

Interestingly, three of these viruses have AUG as an initiator codon of the TGB3 gene. However, in two of these viruses, the initiators have C residues in the −3 and +1 positions, making these contexts non-optimal (Figure 3), suggesting that translation initiation efficiency on these codons is very low and likely comparable to that of the ShVX TGB3 CUG initiator codon. The third virus, *Vanilla latent virus*, has the TGB3 initiator in the suboptimal context GGCAUGU (Figure 3) that would make translation of the downstream p42 gene rather inefficient. Alternatively, translation initiation at this codon may be suppressed due to local structural features of this RNA template. Four of the analyzed viruses have internal AUG triplets in TGB1, TGB2, or both TGB1 and TGB3 genes (Figure 3). In *Blackberry virus E* (BVE), only the reference virus genome used in the analysis has an internal AUG in the TGB2 gene, while all other 19 BVE accessions available in GenBank have non-AUG triplets in this position (Supplementary Table S1). This observation shows that BVE has a ShVX-like arrangement of initiator codons in the TGB/p42 region. Out of the 16 *Garlic virus C* (GarV-C) accessions available, only four, including the reference genome, have internal AUGs in the TGB1 gene. Similarly, only 16 out of 70 *Garlic virus D* (GarV-D) accessions have internal AUG triplets in the TGB1 gene (Supplementary Table S1), all having pyrimidines in −3 and +1 positions, making them rather inefficient initiators. Therefore, the majority of BVE, GarV-C, and GarV-D accessions meet the requirements for p42 translation via the leaky scanning mechanism. The *Garlic virus X* (GarV-X) genome is a notable exception among allexiviruses. Of 45 accessions available in GenBank, only six have no internal AUG triplets in the TGB1 and TGB3 genes. The others have up to two internal AUGs in TGB1 and up to three in TGB3 (Supplementary Table S1), suggesting that the p42 translation mechanism in GarV-X may differ from that of ShVX. In general, based on the sequence analysis, we conclude that the vast majority of sequenced allexiviruses share the p42 expression mechanism involving leaky scanning translation of functionally tetracistronic RNA.

### 2.3. RNA Binding Properties of p42

The biochemical properties of ShVX p42 have not yet been studied. To analyze the ability of p42 to bind RNA, recombinant p42 was expressed in *Escherichia coli* cells. To this end, the p42 coding sequence was cloned into a plasmid vector for protein expression. Then, the p42 protein fused to a 6xHis tag was affinity-purified from lysed bacterial cells and renatured by dialysis. The potential of p42 to bind RNA was analyzed in gel shift experiments, in which a certain amount of recombinant p42 was incubated with increasing amounts of RNA; samples were analyzed in an agarose gel.

**Figure 4:**
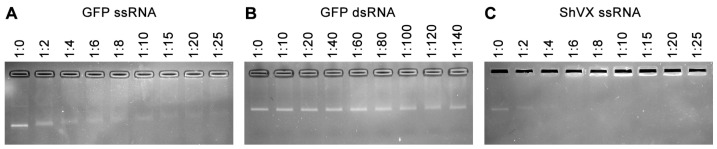
Analysis of the ability of p42 to bind RNA in gel-shift experiments. The binding of p42 to GFP-specific ssRNA (**A**), GFP-specific dsRNA (**B**), and ShVX-specific ssRNA (**C**) is shown. RNA was incubated with increasing amounts of p42 and loaded onto an ethidium bromide-containing agarose gel. The RNA:protein molar ratios are indicated above each lane.

Incubation of p42 with ssRNA, which represented a fragment of the GFP coding sequence, resulted in the formation of RNA–protein complexes with reduced mobility in the gel compared to the non-bound RNA (Figure 4A). These complexes first appeared at an RNA:protein molar ratio of 1:2. As this ratio increased, the mobility of the complexes decreased. Additionally, complexes that were fully retarded and unable to enter the gel were observed at an RNA:protein ratio of 1:10 or higher (Figure 4A). Incubation of p42 with GFP-specific double-stranded RNA (dsRNA) revealed no RNA binding, even at an RNA:protein ratio of 1:140 (the highest ratio tested; Figure 4B). These data demonstrate that p42 binds to ssRNA and that this binding cannot be attributed solely to electrostatic interactions with negatively charged nucleic acids.

Next, the binding of p42 to the ssRNA transcript corresponding to 682 5′-terminal residues of the ShVX genomic RNA was analyzed. Similar to the experiments with GFP-specific ssRNA, both complexes with decreased mobility in the gel and fully retarded complexes were observed (Figure 4C). The binding patterns observed for GFP- and ShVX-specific transcripts were generally similar, with complexes formed by p42 being detected at an RNA:protein molar ratio of 1:2 for both RNAs (Figure 4A,C). However, the fully retarded complexes were observed at slightly lower RNA:protein molar ratios in the case of the ShVX transcript (Figure 4A,C). We assume that the observed differences in binding of GFP- and ShVX-specific transcripts are quantitative. Therefore, these experiments do not reveal any specificity of p42 ssRNA toward the ShVX genome fragment used. Based on the currently available data, we conclude that p42 binds to ssRNA, but not to dsRNA.

### 2.4. Subcellular Localization of p42

**Figure 5:**
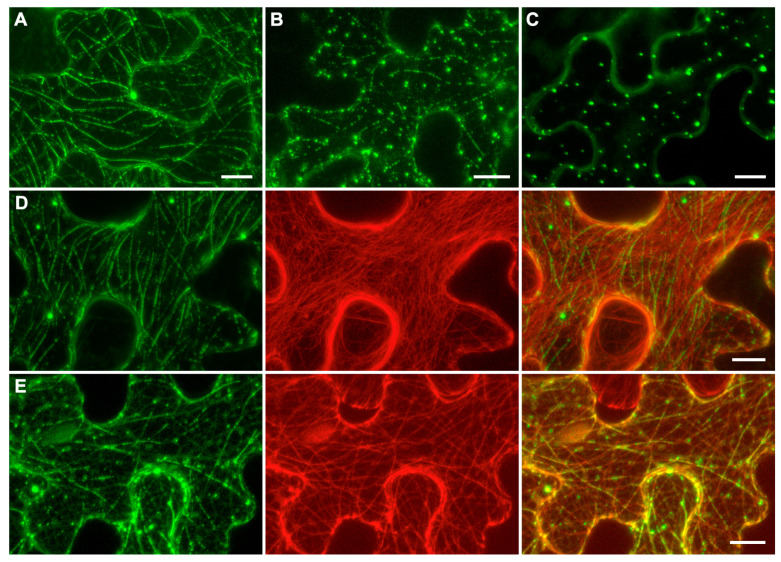
Subcellular localization of p42. (**A**–**C**) Localization of p42-GFP. (**D**) Coexpression of p42-GFP with mRFP-fused C-terminal actin-binding domain of mouse talin. (**E**) Coexpression of p42-GFP with mRFP-fused microtubule-binding domain of mouse MAP4. In (**D**,**E**), left images represent GFP channel, center images—mRFP channel, right images—superposition of images for GFP and mRFP channels. Images were taken at 3 dpi. All images are Z-stacks of optical sections. Scale bar, 10 μm.

To analyze the subcellular localization of ShVX p42, the construct p42-GFP was generated, which encodes a fusion protein with GFP fused to the C-terminus of p42. *N. benthamiana* leaves were agroinfiltrated for expression of p42-GFP, and subcellular localization of the fusion protein was examined using confocal laser scanning microscopy. At 3 dpi, the fusion protein exhibited a diffuse localization in the cytoplasm, localization to thread-like structures in the cytoplasm, and association with small cytoplasmic bodies; these three types of localization were presented to different extents in each particular cell (Figure 5A,B). In a proportion of cells, p42-GFP was found only in numerous bodies dispersed in the cytoplasm (Figure 5C).

The thread-like structures observed for p42-GFP resembled elements of the cytoskeleton. Therefore, *N. benthamiana* leaves were agroinfiltrated for coexpression of p42-GFP with markers of either actin filaments or microtubules. When p42-GFP was coexpressed with the mRFP-fused C-terminal actin-binding domain of mouse talin, known to bind actin microfilaments in plant cells [41], colocalization of GFP and mRFP signal was not observed (Figure 5D). When p42-GFP was coexpressed with the mRFP-fused microtubule-binding domain of mouse MAP4 (microtubule-associated protein 4) [42], the p42-GFP-containing thread-like structures were perfectly colocalized with microtubules (Figure 5E). It should be noted that small bodies containing p42-GFP were also associated with microtubules. Interestingly, not all microtubules in the cell were bound by p42-GFP. In addition, p42 often covered microtubules nonuniformly, forming discontinuous structures that resembled dashed lines (Figure 5A,B) that were aligned with the microtubules (Figure 5E). Therefore, subcellular localization studies show that ShVX p42 can associate with microtubules in plant cells under transient expression conditions. However, it should be kept in mind that, in natural ShVX infection, the p42 localization may be affected by other viral proteins.

### 2.5. Analysis of p42 Ability to Suppress RNA Silencing

Analysis of p42 ability to suppress silencing was carried out using several experimental approaches.

**Figure 6:**
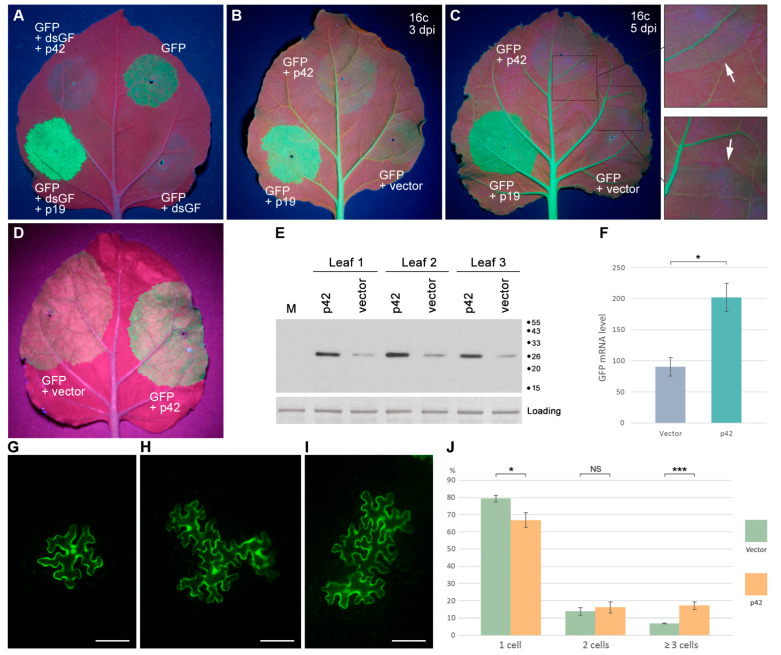
Analysis of p42 ability to suppress RNA silencing. (**A**) Analysis of the ability of p42 to suppress dsRNA-induced silencing. *N. benthamiana* leaves were agroinfiltrated for expression of GFP and coexpression of GFP with dsGF, or dsGF and p19, or dsGF and p42, as indicated. The leaf was imaged at 4 dpi. (**B**,**C**) Analysis of the ability of p42 to suppress ssRNA-induced silencing in transgenic plants. Leaves of transgenic GFP-expressing *N. benthamiana* plants (line 16c) were agroinfiltrated for coexpression of GFP with either p42, p19, or an empty vector. Leaves were imaged at 3 dpi (**B**) and 5 dpi (**C**). Arrows in the enlarged leaf regions indicate the red borders surrounding the infiltrated areas. (**D**–**F**) Analysis of the ability of p42 to suppress ssRNA-induced silencing in non-transgenic plants. (**D**) Leaf agroinfiltrated for coexpression of GFP with either p42 or an empty vector as indicated and imaged under UV light at 3 dpi. (**E**) Western blot analysis of GFP accumulation in leaf areas agroinfiltrated for coexpression of GFP with either p42 or an empty vector. GFP-specific antibodies were used. M, mock-inoculated leaf. The positions of the molecular weight markers are shown on the right. (**F**) Accumulation levels of GFP mRNA in leaves agroinfiltrated for coexpression of GFP with either p42 or an empty vector. Samples were collected at 3 dpi. Average expression levels determined by qPCR are shown; error bars indicate the standard error. Four biological replicates were used to calculate each value shown. The asterisk indicates a statistically significant difference (*, *p* < 0.05) according to the paired two-tailed Student’s *t*-test. (**G**–**J**) Analysis of p42 ability to suppress RNA silencing in TCV-based assay. (**G**–**I**) Representative fluorescent microscopy images of infection loci consisting of TCV-GFP-infected cells. Single-cell (**G**), three-cell (**H**), and four-cell (**I**) loci are shown. Scale bar, 100 μm. (**J**) The graph depicting percentage of the fluorescent loci consisting of one, two, and three or more cells observed upon coexpression of TCV-GFP with either p42 or an empty vector. Asterisks indicate statistically significant (*, *p* < 0.05; ***, *p* < 0.001) differences according to the paired two-tailed Student’s *t*-test. NS, non-significant difference. The error bars indicate the standard error (SE).

First, a previously described experimental system for the identification of viral VSRs was used, which is based on agrobacteria-mediated transient coexpression of GFP and dsGF, a construct containing an inverted repeat of a GFP coding sequence fragment and therefore directing the synthesis of a GFP-specific dsRNA, which initiates efficient silencing of coexpressed full-length GFP [43]. *N. benthamiana* leaves were agroinfiltrated for expression of GFP, dsGF, p42, and p19, a well-characterized *Tomato bushy stunt virus* VSR [44,45] used as a positive control. As revealed under UV illumination at 4 dpi, leaf patches infiltrated for GFP expression demonstrated a moderate level of GFP fluorescence, while coexpression of GFP with dsGF resulted, as expected, in the absence of GFP fluorescence in the infiltrated patches (Figure 6A). When GFP was coexpressed with both dsGF and p19, a high level of GFP fluorescence was observed due to the suppression of dsGF-induced silencing, as described previously [43], whereas coexpression of p42 with GFP and dsGF resulted in no GFP fluorescence (Figure 6A). These observations show that p42 is unable to suppress silencing directed by dsRNA.

Second, the ability of p42 to suppress silencing induced by ssRNA was studied. In these experiments, transgenic GFP-expressing *N. benthamiana* plants of line 16c [46] were used. Leaves of 16c plants were agroinfiltrated for the expression of GFP, p42, and p19. At 3 dpi, observations of infiltrated leaves revealed that GFP coexpressed with a vector (a control) gave a weak GFP signal in the infiltrated areas, whereas coexpression of GFP and p19 resulted in a bright GFP fluorescence (Figure 6B), showing that the p19 VSR activity inhibited silencing of transiently expressed GFP. Compared to coexpression of GFP with a vector, coexpression of GFP with p42 did not result in a significant increase in the level of GFP fluorescence Figure 6B) or a marked increase in the GFP levels, as determined by Western blotting with GFP-specific antibodies (Supplementary Figure S2). At 5 dpi, the relative levels of fluorescence in the infiltrated patches were the same as at 3 dpi; however, a red border surrounding the infiltrated areas appeared in the case of GFP and coexpression of GFP and p42, but not in the case of coexpression of GFP and p19 (Figure 6C). This 10–15 cell-wide border is known to result from the transport of the silencing signal from the infiltrated areas to the surrounding cells, where silencing of the GFP transgene is established [47]. Moreover, p19 is shown to inhibit the cell-to-cell transport of the silencing signals [47]. Taken together, these data demonstrate that p42 has no obvious effect on RNA silencing initiated by ssRNA in transgenic plants or on the spread of silencing from cell to cell.

The ability of p42 to suppress silencing induced by ssRNA was additionally analyzed using another, previously described experimental approach to the identification of the VSR function of a protein [48]. Leaves of *N. benthamiana* plants were agroinfiltrated for coexpression of GFP with either p42 or an empty vector used as a control. As found under UV light at 3 dpi, the p42-expressing areas exhibited brighter GFP fluorescence than control areas (Figure 6D). Western blot analysis with GFP-specific antibodies confirmed higher levels of GFP accumulation upon coexpression with p42 (Figure 6E). qPCR revealed that the level of GFP mRNA was 2.2-fold higher compared to the control (Figure 6F). These data show that the observed p42-induced elevated GFP expression is due to the increased level of GFP mRNA, suggesting that p42 can have the VSR function.

Third, we analyzed the ability of p42 to suppress silencing induced in the context of virus infection using the modified TCV genome, in which the gene of the capsid protein, also exhibiting the VSR activity, has been replaced by the GFP gene. As previously demonstrated, the TCV-GFP is deficient in cell-to-cell transport and is therefore confined to initially infected cells. However, coexpressed heterologous VSRs can restore the TCV transport between cells, as manifested by the formation of multicellular GFP-containing loci [49,50]. Therefore, *N. benthamiana* leaves were infiltrated with a diluted agrobacterial culture for expression of TCV-GFP in individual leaf cells (Figure 6G) located distantly from each other in combination with non-diluted cultures for coexpression of either p42 or an empty vector. Fluorescent microscopy of the infiltrated leaves at 5 dpi revealed a statistically significant (*p* < 0.001 according to Student’s *t*-test) 2.5-fold increase in the percentage of infection loci consisting of three or more cells (Figure 6H,I) in p42 samples compared to empty vector samples (Figure 6J). However, the percentage of multicellular loci observed for p42 was nevertheless low (17.1%) compared to the previously reported value of 76.6% for p19, a strong VSR [51]. Taken together, the experiments on silencing suppression by p42 suggest that p42 can be considered as a weak VSR active in the context of virus infection or under the conditions of silencing activation by ssRNA.

### 2.6. p42 Suppresses Nonsense-Mediated RNA Decay

**Figure 7:**
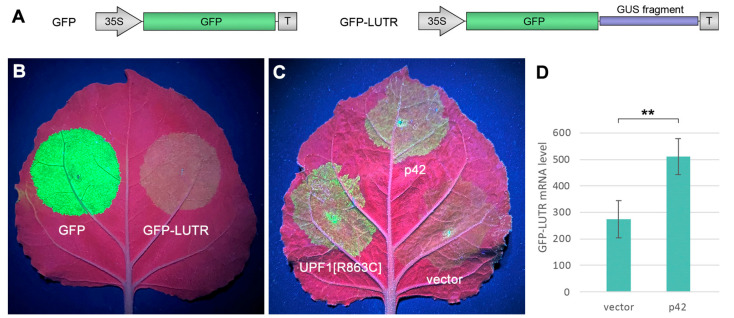
p42 suppresses nonsense-mediated RNA decay. (**A**) Schematic representation of test constructs used. The GFP coding sequence is shown as a green box, and a fragment of the GUS coding sequence is shown as a blue bar. 35S—the CaMV 35S promoter. T—a terminator of transcription. (**B**) GFP-LUTR mRNA undergoes NMD. The leaf was infiltrated with equally diluted agrobacterial cultures for expression of GFP and GFP-LUTR as indicated and imaged under UV light at 4 dpi. (**C**) Analysis of the ability of p42 to suppress NMD. The leaf was agroinfiltrated for coexpression of GFP-LUTR with p42, UPF1[R863C], and an empty vector as indicated and imaged at 4 dpi. (**D**) Accumulation levels of GFP-LUTR mRNA in leaves agroinfiltrated for coexpression of GFP-LUTR with an empty vector and p42. Samples were collected at 4 dpi. Average expression levels determined by qPCR are shown; error bars indicate the standard error. Three biological replicates were used to calculate each value shown. The asterisk indicates a statistically significant difference (**, *p* < 0.01) according to the paired two-tailed Student’s *t*-test.

Like other alpha-like viruses, ShVX employs the synthesis of sgRNAs for the expression of 5′-distal genes in its polycistronic genomic RNA. Consequently, the ShVX genomic RNA and TGB1 sgRNA have long 3′-untranslated regions (3′UTRs) and therefore can be targeted by nonsense-mediated decay (NMD), a cell RNA quality control mechanism that degrades aberrant RNAs, including those with long 3′-UTRs, such as incorrectly spliced mRNAwith internal termination codons [52,53]. Therefore, we analyzed whether p42 can suppress the NMD-based antiviral strategy in plants. For these experiments, we used a reporter construct named GFP-LUTR (for “Long UTR”), which contains a 555 nt fragment of the GUS gene placed between the GFP coding sequence and the transcription terminator in a GFP expression vector (Figure 7A). Therefore, the mRNA transcribed from this construct in plants had a long 3′-UTR. Such long 3′UTRs have been shown to act as NMD-activating *cis*-elements in plant cells [54]. To verify whether GFP-LUTR RNA undergoes NMD, leaves of *N. benthamiana* plants were agroinfiltrated for the expression of either GFP-LUTR or GFP, which was used as a control. In these and subsequent experiments, the test constructs were coexpressed with p14, a potent VSR of *Pothos latent virus*, to analyze the NMD effect under conditions when RNA silencing is suppressed [54].

As expected, observations of infiltrated leaves under UV light at 4 dpi revealed that the GFP-LUTR fluorescence was considerably decreased compared to that of GFP (Figure 7B). To test the ability of p42 to suppress NMD, *N. benthamiana* plants were agroinfiltrated for the coexpression of GFP-LUTR with p42, an empty vector (a negative control), or UPF1[R863C] (a positive control). The latter construct contained a cloned sequence of *Arabidopsis thaliana* UPF1 (RNA helicase Up-Frameshift 1), the core effector of NMD [52], carrying a point mutation of arginine to cysteine residue in position 863. This UPF1 mutant is known to be dominant-negative upon transient expression and capable of suppressing NMD in plant cells [53,54]. As observed under UV light at 4 dpi, coexpression of UPF1[R863C] with GFP-LUTR increased the level of GFP fluorescence compared to coexpression of GFP-LUTR with the empty vector (Figure 7C), confirming, therefore, that GFP-LUTR mRNA undergoes NMD. Coexpression of p42 with GFP-LUTR also increased the fluorescence level compared to the negative control (Figure 7C). Analysis by qPCR revealed that the level of GFP-LUTR RNA was increased 1.86-fold in the presence of p42 (Figure 7D). These data show that p42 is able to suppress NMD.

## 3. Discussion

To date, the expression mechanism and functions of ShVX p42, an orphan protein that exhibits no sequence similarity to known viral or cellular proteins, have remained unstudied. The analysis of ShVX gene expression, as reported in this paper, shows that a p42-specific sgRNA is not detected in infected shallot plants. As found in experiments on the transient expression of the cloned TGB/p42 genomic region in *N. benthamiana* plants, p42 can be translated via the leaky scanning mechanism on the template of tetracistronic RNA that contains TGB1, TGB2, TGB3, and p42 genes. As the CP gene, located right downstream of the p42 gene, is expressed via its own sgRNA, we assume that the model tetracistronic RNA corresponds to TGB1 sgRNA in terms of its coding capacity. Therefore, we suggest that the TGB1 sgRNA serves as the template for p42 translation in natural ShVX infection. The 5′-UTR of TGB1 sgRNA is only three nt long, as found by 5′-RACE. This finding agrees well with the data showing that such extremely short 5′-UTRs in TGB1 sgRNAs of other viruses inhibit TGB1 translation initiation, promote leaky scanning, and, thus, are essential for the efficient translation of downstream genes [36]. As demonstrated by mutagenesis, the ShVX p42 expression via leaky scanning requires the TGB2 initiator codon to be in a suboptimal context and TGB3 translation to be initiated with low efficiency at an alternative initiator codon (CUG). In addition, the entire ShVX TGB region lacks AUG triplets, except for the TGB1 and TGB2 initiator codons, enabling scanning ribosome subunits that bypass the TGB2 and TGB3 initiators to reach the p42 initiator codon. These features are present in all other known allexiviruses, with one exception, suggesting that the p42 expression mechanism described here for ShVX is conserved in the *Allexivirus* genus.

The synchronized expression of p42 and TGB from a single sgRNA indicates that these four genes constitute a single genomic module. This suggestion is consistent with the previously published observation that the accumulation of mutations in ShVX persisted in vegetatively reproduced shallot plants for twenty years was markedly lower in the TGB/p42 genome region than in other parts of the genome [55], suggesting that the TGB/p42 region evolved as a separate genome unit. Hypothetically, p42 may be functionally related to the TGB proteins, playing an accessory role in virus cell-to-cell transport mediated by TGB proteins. In this regard, it is worth mentioning that p42 is found to colocalize with microtubules in plant cells, making a parallel to the transient association of the 30-kDa TMV MP with microtubules early in infection [56]. It is tempting to speculate that p42 has a movement-related function. However, there are currently no experimental data to support this hypothesis, and its experimental validation is the subject of our further investigations.

The data presented in this paper suggest that p42 functions are related to RNA metabolism in plant cells. In transient expression assays, p42 is shown to suppress RNA silencing induced by ssRNA, but not dsRNA. This finding correlates well with the results of in vitro experiments in which p42 is found to efficiently bind ssRNA, but exhibits no dsRNA binding. Silencing of ssRNA, such as transcripts of transgenes, involves the RNA-dependent RNA polymerase RDR6, which converts ssRNA into dsRNA, a substrate for DICER-like (DCL) proteins that dice dsRNA into small interfering RNAs (siRNA) targeting the initial ssRNA for degradation by Argonaute (AGO) proteins [57,58]. The majority of protein-coding mRNA in plant cells is believed to be protected from this type of degradation by 5′-cap-binding and poly(A)-binding proteins, which inhibit the access of RDR6 and other factors involved in siRNA biogenesis [59,60]. According to the threshold hypothesis, mRNA of transgenes can be degraded by this pathway when transcript levels exceed the capacity of available cap- and poly(A)-binding proteins to protect mRNA from siRNA biogenesis machinery [59,60]. We hypothesize that the observed inhibition of ssRNA-induced silencing by p42 can be attributed to the p42 ssRNA binding, which inhibits access of RDR6 and/or other silencing factors to transiently expressed transgene RNA. This model is consistent with the high efficiency of ssRNA binding by p42. In fact, at an RNA:protein molar ratio of 1:2, that is at the ratio of one ssRNA molecule per one p42 dimer, all RNA appeared to be bound by p42. The inability of p42 to bind dsRNA and to inhibit silencing induced by dsRNA can suggest that p42 may protect ssRNA from RDR6, but not from dsRNA-derived siRNA. It remains to be established why p42, upon transient coexpression with GFP, is able to increase the level of GFP expression in non-transgenic *N. benthamiana* plants, but not in GFP-expressing transgenic plants. We assume that the expression of the GFP transgene in these plants is partially suppressed by post-transcriptional silencing, and that p42 cannot protect the transiently expressed GFP mRNA from silencing directed by pre-existing GFP-specific siRNAs.

In some instances, viral VSRs, in addition to their main function, can suppress other types of plant defense aimed at the degradation of viral RNA. For example, HC-Pro and VPg, the two VSRs of *Turnip mosaic virus*, have been shown to bind to and inhibit key proteins involved in an exonuclease-mediated RNA decay, the mechanism by which RNA is degraded when the post-transcriptional silencing is suppressed [61]. In this paper, we demonstrate that ShVX p42, in addition to its VSR function, can suppress NMD, the cell RNA quality control mechanism that targets for degradation, in particular, aberrant mRNAs with unusually long 3′-UTRs [52] and polycistronic RNAs of plant alpha-like viruses [53]. Similarly, TCV CP has been shown to suppress both RNA silencing [18] and NMD [62]. Other plant virus proteins known to suppress NMD are the *Cauliflower mosaic virus* TAV protein, which targets the decapping machinery through the scaffold protein VARICOSE [63], and the *Pea enation mosaic virus 2* p26 protein, which is involved in systemic transport of viral RNA through the phloem and inhibits NMD via an unknown mechanism [64]. Further experiments are required to determine the p42-specific mechanism of NMD suppression and its relationship to the p42 VSR activity.

Many plant virus MPs also have the VSR function [65]. One such MP is the TGB1 protein, for which the roles of the VSR and transport functions in virus cell-to-cell transport have been thoroughly investigated in viruses of the *Alphaflexiviridae* and *Betaflexiviridae* families. In a pioneering work by Bayne et al. [66], the transport function of some movement-deficient point mutants of PVX TGB1 protein has been shown to be complemented by coexpression of a heterologous VSR, TBSV p19, showing that suppression of RNA silencing is essential for TGB1 to function as an MP. A point mutation in the *Alternanthera mosaic virus* TGB1 protein has been shown to considerably inhibit the VSR function, while leaving the transport function unaffected [67], demonstrating that the suppression and transport functions can be uncoupled. Thus, TGB1 has two functions essential for virus cell-to-cell movement, the VSR function and the transport function per se. It is currently unknown whether ShVX TGB1 can serve as a VSR by suppressing silencing signal transport, inhibiting RDR6/SGS3 activity, or directing the degradation of AGO proteins, as demonstrated for TGB1 of other viruses [48,68,69]. We hypothesize that ShVX p42, as an additional component of the TGB-containing gene module, provides the silencing suppression function that may be required for efficient TGB-mediated virus cell-to-cell transport. The p42 VSR function can replace the TGB1 VSR function known for other viruses; alternatively, p42 can act as an additional VSR together with the functional TGB1 VSR. Such a dual silencing suppression strategy has been described for *Potato virus M*, in which TGB1 and CRP are two viral VSRs with different functions: while TGB1 only suppresses the spread of silencing signals, CRP acts as a VSR at the levels of both virus replication and the spread of silencing signals [48]. Therefore, we propose that the ShVX p42 gene and TGB constitute a single genomic module encoding four proteins, together involved in virus cell-to-cell transport and RNA silencing suppression. Another such module described as “tetra-cistron movement block” (TCMB) has been identified in the transcriptomes of the flowering plant *Colobanthus quitensis* and the moss *Dicranum scoparium* [70]. Similar to the ShVX TGB/p42 gene block, TCMB consists of four genes, namely, TGB and a gene that encodes vDRB, an RNA-binding protein exhibiting the VSR properties [71]. Unlike p42, however, vDRB binds dsRNA rather than ssRNA, and the vDRB gene is located upstream of TGB [70,71]. Thus, TCMB and the ShVX TGB/p42 gene block are the two examples of “extended” TGB that have evolved independently and are dissimilar. However, in both viruses, the “extensions”, or additional genes, provide TGB with the VSR function. The presence of additional VSR-encoding genes in two TGB-related transport gene modules emphasizes the necessity of silencing suppression for TGB-mediated movement of viruses from cell to cell.

Taken together, the data presented in this paper demonstrate that the ShVX TGB/p42 gene module is a single genomic unit in terms of protein expression. The suppressor activity of p42 is relatively weak compared with well-characterized VSRs such as p19, and its biological significance in the context of ShVX infection remains to be determined. The observed colocalization with microtubules raises the possibility of an accessory role of p42 in virus movement; however, this remains a hypothesis requiring direct experimental validation. The results reported here provide a foundation for future functional analyses of ShVX p42 aimed at determining the quantitative parameters of RNA binding, assessing potential synergy with other ShVX proteins, expanding the characterization of NMD suppression capabilities, and clarifying the role of p42 in the natural shallot host.

## 4. Materials and Methods

### 4.1. Primers

Primers used in this study for molecular cloning, mutagenesis, qPCR, and 5′-RACE are listed in Supplementary Table S2.

### 4.2. Recombinant Constructs

The recombinant constructs for the transient expression of mRFP-fused C-terminal actin-binding domain of mouse talin [41], mRFP-fused microtubule-binding domain of mouse MAP4 [42], TCV-GFP [49,50], and GFP and dsGF [43] were described previously. The recombinant construct pBSCII-SK(+)-GFP was described previously [72].

Coding region of the p14 protein of *Pothos latent virus* was chemically synthesized (Evrogen, Moscow, Russia) according to previously published sequences [73,74] and cloned into the binary vector pLH* [75] to give pLH-p14, respectively.

To obtain the TGB-p42-FLAG construct, the corresponding region of the ShVX genome was amplified by overlap PCR on the template of cDNA from ShVX-infected *Allium cepa var. aggregatum* plants with specific primers. The first PCR product was obtained using primers p1-Xh-P/ShVX-p1-ovl-M. The second product was obtained with primers ShVX-p1-ovl-P/p42-Xba-M. The resulting overlap amplification product was amplified using p1-Xh-P/p42-Xba-M primers and then digested with *Xho*I-*Xba*I (ThermoFisher Scientific, Waltham, MA, USA) restriction endonucleases and cloned into the pLH* binary vector.

For the TGB2[OPT] and TGB2[CAG] constructs, the corresponding sequences were amplified by overlap PCR with appropriate primers on the TGB-p42-FLAG construct template. The first PCR product was obtained with Left/ShVX-ovl-M primers on the TGB-p42-FLAG template. The second fragment was amplified on the same template with ShVX-ovl-P-p2CAG/ShVX-p2-Spe-M or ShVX-ovl-P-p2opt/ShVX-p2-Spe-M primer pairs. The final overlap PCR product was amplified with Left/ShVX-p2-Spe-M primers, digested by *Xho*I-*Spe*I restriction endonucleases, and cloned into the TGB-p42-FLAG construct to replace the wild-type sequence.

Similarly, the first fragment of both TGB3[AUG] and TGB3[CAG] constructs was obtained by PCR with Left/ShVX-BglII-M primers on the TGB-p42-FLAG template and digested by *Xho*I-*Bgl*II restriction endonucleases (ThermoFisher Scientific, Waltham, MA, USA). The second fragment was amplified by overlap PCR on the same template with ShVX-BglII-P/ShVX-p3AUG-ovl-M or ShVX-BglII-P/ShVX-p3CAG-ovl-M primer pairs for the first part of the overlap PCR and ShVX-p3-ovl-P/Right primer pair for the second. The final overlap PCR product was amplified with ShVX-BglII-P/Right primers and digested by *Bgl*II-*Xba*I restriction endonucleases. The digested PCR products for the first fragment and the overlap PCR product were then cloned into pLH* at the *Xho*I-*Xba*I sites.

The construct of non-fused p42 in pLH* (pLH-p42) was obtained by amplifying two parts of the p42 coding sequence using p42-P-XhoNhe/p42-intEco-M and p42-intEco-P/p42-M-SalXba primer pairs on a ShVX cDNA template, followed by digestion with *Xho*I-*Eco*RI and *Eco*RI-*Xba*I restriction endonucleases (ThermoFisher Scientific, Waltham, MA, USA), respectively. The resulting products were then cloned into the pLH* binary vector at the *Xho*I-*Xba*I sites.

For the p42-GFP construct, the p42 coding sequence was amplified on pLH-p42 with p42-P-XhoNhe and p42-M-Bam primers, then digested by *Xho*I-*Bam*HI endonucleases (ThermoFisher Scientific, Waltham, MA, USA) and subcloned as a C-terminal fusion with GFP into pLH-BMB1-GFP [76], from which the BMB1 sequence was removed using the *Xho*I-*Bam*HI restriction sites.

The p42 coding sequence was amplified with primers p42-pET-M-Xho and p42-P-XhoNhe using a previously obtained pLH-p42 construct serving as a template. The resulting PCR product was digested with *XhoI*-*NcoI* and ligated into similarly digested vector pET-33b(+) (*Novagen*, *Darmstadt*, *Germany*).

To obtain the UPF1[R863C] construct, the UPF1 gene was amplified on an *Arabidopsis thaliana* cDNA template by overlap PCR with appropriate primers. The first amplification product was obtained using primers UPF1-Xho-P/UPF1-ovl-M and UPF1-ovl-P/UPF1-Xba-M primers in the case of the second product; the resulting overlap product was amplified with UPF1-Xho-P/UPF1-Xba-M primers, cut with restriction endonucleases *Xho*I––*Xba*I (ThermoFisher Scientific, Waltham, MA, USA), respectively, and cloned into pLH* binary vector.

The GFP-LUTR construct was obtained by ligation of two PCR products into the binary vector pLH*. At the first step, appropriate PCR products were amplified with Left/C3-Mlu-M primers on the pLH*-GFP template [43] and pLH*-GUS template [75] with GUS2-P120/GUSi-M primers, respectively. In a second step, PCR products were digested with *Nco*I-*Mlu*I and *Mlu*I-*Xba*I restriction endonucleases (ThermoFisher Scientific, Waltham, MA, USA) and cloned into the binary vector pLH* digested with *Nco*I-*Xba*I restriction endonucleases (ThermoFisher Scientific, Waltham, MA, USA).

### 4.3. 5′-RACE

The 5′-RACE was carried out using the Ambion FirstChoice RLM-RACE Kit (ThermoFisher Scientific, Waltham, MA, USA) according to the manufacturer’s protocol.

### 4.4. Protein Expression and Purification

*E. coli* cells (strain BL21) were transformed with the expression vector pET-p42, and clones with the highest expression levels were selected. Bacterial cells were grown in 2 YT medium in the presence of kanamycin (25 μg/mL) at 37 °C until an optical density at 600 nm (OD_600_  =  0.8) was reached. To induce protein expression, IPTG was added to a final concentration of 1 mM, and the cultures were incubated at 37 °C for 3 h. Cells were pelleted at 4500 g for 10 min. The recombinant protein carrying the N-terminal 6xHis tag was purified by denaturing Ni-NTA chromatography according to the Ni-NTA agarose manufacturer’s instructions (Qiagen, Hilden, *Germany*). Purified protein was analyzed by SDS electrophoresis in a 15% polyacrylamide gel according to Laemmli and renatured by dialysis against renaturation buffer (5 мM Tris-HCl, DTT 1 mM, 5% glycerol, pH = 7, 8) at room temperature. The final protein concentration was measured using UV absorption spectrometry.

### 4.5. Gel-Shift Experiments

RNA fragments used in gel-shift experiments were obtained by in vitro transcription with T7 RNA polymerase (ThermoFisher Scientific, Waltham, MA, USA). The ssRNA GFP substrate was obtained by transcription of the pBSCII-SK(+)-GFP linearized with *Sac*I restriction endonuclease. The dsRNA GFP substrate was synthesized by transcription of a PCR product obtained on the template of pBSCII-SK(+)-GFP with a pair of T7 promoter-containing primers dsC3-P and dsC3-M.

To obtain the ssRNA ShVX substrate, first, total RNA was extracted from ShVX-infected plants using ExtractRNA reagent (Evrogen, Moscow, Russia) according to the manufacturer’s instructions. The total RNA sample was transcribed into cDNA with ShVX-M-XAN primer using a Revertaid reverse transcriptase (ThermoFisher Scientific, Waltham, MA, USA). The resulting cDNA was then used as a template for PCR with primers ShVX-M-XAN and ShVX-T7-P. Finally, ssRNA ShVX was synthesized via transcription of the resulting PCR product.

In gel-shift experiments, nucleic acids were incubated with variable amounts of a protein in a binding buffer (10 mM Tris-HCl pH 7.8, 50 mM KCl, 0.1 mM EDTA, 5% Glycerol) for 30 min on ice. The samples were analyzed in a 1.8% non-denaturing agarose gel containing ethidium bromide.

### 4.6. Plant Material

*Nicotiana benthamiana* plants were grown and maintained under standard conditions (16 h/8 h light/dark cycles, 24/20 °C day/night temperatures, 50% humidity) in growth chambers. Five- to six-week-old plants were used for agroinfiltration for transient gene expression.

### 4.7. Plant Agroinfiltration

*Agrobacterium* tumefaciens strain C58C1 cells were transformed with binary vectors using the freeze–thaw method. Agroinfiltration of transformed *A. tumefaciens* cell cultures was preceded by an overnight growth at 28 °C with shaking at 175 rpm in liquid Luria-Bertani (LB) medium, with the necessary antibiotic selectivity. The cells were then pelleted by centrifugation, resuspended in infiltration buffer (10 mM MES, pH 5.5; 10 mM MgCl_2_; 150 μM acetosyringone), and incubated for 3 h at room temperature. For infiltration, the cultures were diluted to a final optical density of 0.3 at 600 nm (OD_600_ = 0.3). Agrobacterium culture mixtures were infiltrated into the upper, fully formed leaves of *N. benthamiana* plants via the abaxial epidermis using a needle-less syringe.

### 4.8. Western Blot Analysis

Samples of *N. benthamiana* leaf tissue were frozen and ground into a powder using liquid nitrogen. Then, two parts of a 250 mM Tris-HCl buffer solution (pH 7.8) and one part of a 4× Laemmli sample buffer solution (100 mM Tris-HCl, pH 6.8; 100 mM β-mercaptoethanol; 10% glycerol; 4% SDS; 0.1% bromophenol blue) were added to one part of the sample. The mixture was then incubated for five minutes at 95 °C, after which the supernatant was collected from the plant residues by centrifugation and loaded onto 12% polyacrylamide SDS-PAGE. Proteins from the gel were transferred to a Hybond-P membrane (GE Healthcare Bio-Sciences, Niskayuna, NY, USA). Protein detection was conducted with peroxidase-conjugated rabbit Anti-GFP antibodies (Rockland, Pottstown, PA, USA) or monoclonal mouse Anti-FLAG M2 primary antibodies (Sigma-Aldrich, Saint Louis, MO, USA) and rabbit Anti-Mouse IgG (whole molecule)-Peroxidase secondary antibodies (Sigma-Aldrich, Saint Louis, MO, USA) and the ECL system (GE Healthcare Bio-Sciences).

### 4.9. Quantitative PCR

For qPCR analysis, plant tissue was collected and frozen in liquid nitrogen. RNA was isolated using the ExtractRNA reagent (Evrogen, Moscow, Russia), following the manufacturer’s instructions. The RNA samples were then thoroughly treated with RNase-free DNase I (ThermoFisher Scientific, Waltham, MA, USA). For reverse transcription, 2 μg of RNA from each sample was transcribed into cDNA with oligo(dT) primer; the reaction was carried out using M-MuLV Reverse transcriptase (SibEnzyme, Novosibirsk, Russia). The reverse transcription product was diluted fivefold for qPCR. The qPCR reaction was performed on a CFX Connect Real-Time PCR System (Bio-Rad, Hercules, CA, USA) using qPCRMix M-440 (Synthol, Moscow, Russia). A pair of sequence-specific primers, GFP-C3-PP1-R and GFP-C3-PP1-F, was used for GFP and GFP-LUTR mRNA detection, as well as a pair of F-box-F and F-box-R primers for the reference F-box protein mRNA. The Ct value for the GFP and GFP-LUTR mRNA was normalized to the F-box protein reference gene mRNA. The results were statistically analyzed using a paired-samples *t*-test.

### 4.10. Microscopy and Visualization of Fluorescence

Agroinfiltrated leaf areas were analyzed from the abaxial leaf side at 3 dpi. Subcellular protein localization was analyzed using a Nikon C2plus confocal laser scanning microscope (Tokyo, Japan), equipped with a ×60 (1.2 NA) water immersion objective. The fluorescence excitation wavelengths were 488 nm for GFP and 548 nm for mRFP. The acquisition bands for GFP were 495–545 nm, while those for mRFP were 580–640 nm. In the Z-series of optical sections, successive images were captured at 2 µm intervals, and the images were processed using Nikon NIS Elements software (version 5.21.00). GFP fluorescence in infiltrated leaves and 16c plants was excited under UV-A light (365 nm) using UV-365ES UV-A/White Light LED NTD Inspection Lamp (Spectro-UV, Farmingdale, NY, USA).

### 4.11. TCV-GFP Assay

A TCV-based assay, as described earlier [49], was used to detect VSR activity. Briefly, prior to agroinfiltration, bacterial cultures carrying p42, empty vector control, or p19 constructs were diluted to OD_600_ = 0.3. The TCV-GFP-containing culture was diluted 1250-fold more to a final OD_600_ = 0.00024 in order to obtain the individually transformed cells. The abaxial side of young leaves was infiltrated with the tested combination of agrobacteria with a 2 mL needle-less syringe. The results of the TCV-GFP-based essay were examined at 5 dpi. The size of TCV-derived loci was analyzed using a Zeiss Axiovert 200 M epifluorescent microscope (Zeiss, Oberkochen, Germany) with a ×20 objective. A total of 513 TCV-GFP-infected cell loci were measured for p42, and 486 loci were measured for the vector control. The results were statistically analyzed using a Student’s *t*-test.

### 4.12. Sequence Analysis

Assembled viral genomes were collected from the NCBI database (accessed on 17 June 2025). The detection of ORFs and the translation of putative protein-coding sequences were performed using the ORF Finder program (https://www.ncbi.nlm.nih.gov/orffinder/) and ExPASy (https://web.expasy.org/translate/), respectively. These tools were accessed on 17 June 2025.

## Data Availability

Data are contained within the article and supplementary materials.

## Supplementary Materials

The following supporting information can be downloaded at: https://www.mdpi.com/article/10.3390/plants14162552/s1, Figure S1: Prediction of initiator codons in TGB1 genes; Figure S2: Western blot analysis of GFP accumulation in leaves of transgenic GFP-expressing *N. benthamiana* plants (line 16c) agroinfiltrated for coexpression of GFP with either p42 or an empty vector used as a control; Table S1: Occurrence of internal AUG triplets in TGB/p42 regions of selected allexiviruses; Table S2: Primers used in this study.
