# Supplementary material for: Shallot virus X p42 Protein Expressed in Concert with Virus Movement Proteins Is a Suppressor of Two Plant Antiviral Defense Mechanisms
Source: Plants (Basel). 2025 Aug 16;14(16):2552. doi: 10.3390/plants14162552 (PMC12389679; doi:10.3390/plants14162552)
Supplement: Supplementary file 1 [file plants-14-02552-s001.zip › Supplementary_materials.pdf]

```
ShVX     ACTAATTAACTAACCCCTTAGCATTAGTTATGTTTTATAGGTGTTTGAAATG
BVE      TGATTTGCCACCGGGCCGTTGAGTAGCTTATGTTTTGCCTAAATCTGAAGATG
GarV-A   TCATTTGCTGACAACCTTTGTAGCTAGTTATGTTCTAGATATGCTCGAAATG
GarV-B   CAACTTTAAAGCATCCATATGGTTTAGTTATGTTTCCTAATGTGTTTGAAATG
GarV-C   ATATATATAGAATCCCATGTAAATAGGTTAGGTTTTCCTGCATGATTGAAATG
```

Supplementary Figure S1. Prediction of initiator codons in TGB1 genes. The sequences of BVE, GarV-A, GarV-B, and GarV-C were aligned with the ShVX sequence upstream of the TGB1 initiator codon. The 5’-terminal residue of the TGB1 sgRNA that was mapped for ShVX and predicted for the other viruses is shown in green. The conserved core of the sgRNA promoter is shown in blue. The TGB1 initiator codons are shown in bold. Triplet previously annotated as the initiator codons of the respective genes are underlined.

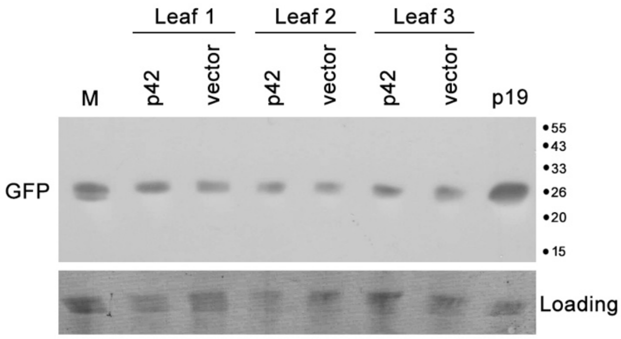

Supplementary Figure S2. Western blot analysis of GFP accumulation in leaves of transgenic GFP-expressing *N. benthamiana* plants (line 16c) agroinfiltrated for coexpression of GFP with either p42 or an empty vector used as a control. M, mock (non-infiltrated leaf area). p19, leaf area agroinfiltrated for coexpression of GFP and p19. The positions of the molecular weight markers are shown on the right.

Supplementary Table S1. Occurrence of internal AUG triplets in TGB/p42 regions of selected allexiviruses.

Virus name	Number of virus accessions in GenBank	Number of accessions with internal AUG between initiators of the genes		
		TGB1 and TGB2	TGB2 and TGB3	TGB3 and p42
BVE	20	0	1	0
GarV-C	16	4	0	0
GarV-D	70	16	0	0
GarV-X	45	35	0	38

Supplementary Table S2. Primers used in this study.

**5´RACE**	
R-TGB1-in	CGTCAAGAATTCTGGTCTCGTGA
R-TGB1-out	GAATGGGTCACCTACAAGGACAT
R-TGB2-in	CGGAATTCTAGTGAGAAGTATTGCCAGGGT
R-TGB2-out	AGGTTGAGAACAGCGTACGCAA
R-p42-in	GCGAATTCGGTTGAGTAGAGCGTTGAAGT
R-p42-out	TTGACGCAATTGGGTTTGGGCT
R-CP-in	ACGGAATTCGACTCCACGTCACTAGTGAT
R-CP-out	CTCTTAGCTTGTAAGGCGTCCA
R-CRP-in	GTCAGAATTCGAGCGACTCGTAAAGCGTT
R-CRP-out	CCCTGATGGATGACACTCAAACA
**TGB-p42-FLAG**	
p1-Xh-P	GGCTCGAGAAATGAAGACTGACCTCCTACT
p42-Xba-M	GCTCTAGAATCACTTGTCATCGTCATCCTTGTAATCACACAGACCTTCGCCCCTAA
ShVX-p1-ovl-M	CTAAGTGCTCGTGTGGTAGCGAT
ShVX-p1-ovl-P	ATCGCTACCACACGAGCACTTAG
**TGB2[OPT] and TGB2[CAG]**	
Left	TATCCTTCGCAAGACCCTTCCTCT
ShVX-ovl-M	GTAACTCCAATGTTGTGTCGGT
ShVX-ovl-P-p2CAG	ACCGACACAACATTGGAGTTACCACCAGAGCTTTGC
ShVX-ovl-P-p2opt	ACCGACACAACATTGGAGTTACACCATGGGCTTTGC
ShVX-p2-Spe-M	GCGACTAGTGAGAAGTATTGCTAGCGTGAGCG
**TGB3[AUG] and TGB3[CAG]**	
Right	CCTTATCTGGGAACTACTCACACATT
ShVX-BglII-M	AAGCCTACAAGATCTCGCTCAAATTC
ShVX-BglII-P	AGCGAGATCTTGTAGGCTTCTAC
ShVX-p3CAG-ovl-M	GAAATGGCTTGTTGGTGATGATCCTGTTTTAGG
ShVX-p3AUG-ovl-M	GAAATGGCTTGTTGGTGATGATCCATTTTTAGG
ShVX-p3-ovl-P	GATCATCACCAACAAGCCATTT
**p42 and p42-GFP**	
p42-P-XhoNhe	CGCTCGAGCTAGCCATGGTGATTGTCACAACCTTCC
p42-intEco-M	CGGAATTCGGGAGTCTACGCCG
p42-intEco-P	CCGAATTCCGATGGACATAACAGGTAGGCCAGCAAGCAC
p42-M-SalXba	GCTCTAGAGTCGACTTAACACAGACCTTCGCCCC
p42-M-Bam	GCGGATCCAGAACACAGACCTTCGCCCCTA
**UPF1[R863C]**	
UPF1-Xho-P	CGCTCGAGACCATGGATTCTCAACAGAGCGATCT
UPF1-Xba-M	CGTCTAGACTTTCTCACCCTCAGCCATTGT
UPF1-ovl-P	ATATTGTCCTGTGTCTGCAGTAATGAGCATCA
UPF1-ovl-M	TGATGCTCATTACTGCAGACACAGGACAATAT
**GFP-LUTR**	
GUS2-P120	GCGACGCGTTACAAGAAAGCCGGGC
GUSi-M	GCTCTAGACCACCTGTTGATCCGCATC
Left	TATCCTTCGCAAGACCCTTCCTCT
C3-Mlu-M	CGCACGCGTTATTTGTAGAGCTCATCCATGCC
**qRCR**	
F-Box-F	GGCACTCACAAACGTCTATTTC;
F-Box-R	ACCTGGGAGGCATCCTGCTTAT
GFP-C3-PP1-R	GCGTCTTGTAGTTCCCGTCA
GFP-C3-PP1-F	TCAGTGGAGAGGGTGAAGGT
**Primers for protein expression and gel-shift experiments**	
p42-pET-M-Xho	CGCTCGAGAGAACACAGACCTTCGCCCCTA
p42-P-XhoNhe	CGCTCGAGCTAGCCATGGTGATTGTCACAACCTTCC
ShVX-M-XAN	CCTGCTAGCGGCGCGCCTGTACACGTCAGGAAATA
ShVX-T7-P	CGTAATACGACTCACTATAGAAAATCAACCAAAACATCACACAACCA
dsC3-P	GAGAATTCTAATACGACTCACTATAGGGTGAAGGTGATGCTACATA
dsC3-M	GAGAATTCTAATACGACTCACTATAGGGCAGATTGTGTCGACAG
